# Dantrolene Prevents the Lymphostasis Caused by Doxorubicin in the Rat Mesenteric Circulation

**DOI:** 10.3389/fphar.2021.727526

**Published:** 2021-08-16

**Authors:** Serena Van, Soumiya Pal, Brittney R. Garner, Kate Steed, Vijayalakshmi Sridharan, Shengyu Mu, Nancy J. Rusch, Amanda J. Stolarz

**Affiliations:** ^1^ Department of Pharmaceutical Sciences, College of Pharmacy, University of Arkansas for Medical Sciences, Little Rock, AR, United States; ^2^ Department of Pharmacology and Toxicology, College of Medicine, University of Arkansas for Medical Sciences, Little Rock, AR, United States

**Keywords:** lymph vessel, lymph flow, dantrolene, calcium signaling, doxorubicin, ryanodine receptor

## Abstract

**Background and Purpose:** Doxorubicin (DOX) is a risk factor for arm lymphedema in breast cancer patients. We reported that DOX opens ryanodine receptors (RYRs) to enact “calcium leak,” which disrupts the rhythmic contractions of lymph vessels (LVs) to attenuate lymph flow. Here, we evaluated whether dantrolene, a clinically available RYR1 subtype antagonist, prevents the detrimental effects of DOX on lymphatic function.

**Experimental Approach:** Isolated rat mesenteric LVs were cannulated, pressurized (4–5 mm Hg) and equilibrated in physiological salt solution and Fura-2AM. Video microscopy recorded changes in diameter and Fura-2AM fluorescence tracked cytosolic free calcium ([Ca^2+^_i_]). High-speed *in vivo* microscopy assessed mesenteric lymph flow in anesthetized rats. Flow cytometry evaluated RYR1 expression in freshly isolated mesenteric lymphatic muscle cells (LMCs).

**Key Results:** DOX (10 μmol/L) increased resting [Ca^2+^_i_] by 17.5 ± 3.7% in isolated LVs (*n* = 11). The rise in [Ca^2+^_i_] was prevented by dantrolene (3 μmol/L; n = 10). A single rapid infusion of DOX (10 mg/kg i.v.) reduced positive volumetric lymph flow to 29.7 ± 10.8% (*n* = 7) of baseline in mesenteric LVs *in vivo*. In contrast, flow in LVs superfused with dantrolene (10 μmol/L) only decreased to 76.3 ± 14.0% (*n* = 7) of baseline in response to DOX infusion. Subsequently, expression of the RYR1 subtype protein as the presumed dantrolene binding site was confirm in isolated mesenteric LMCs by flow cytometry.

**Conclusion and Implications:** We conclude that dantrolene attenuates the acute impairment of lymph flow by DOX and suggest that its prophylactic use in patients subjected to DOX chemotherapy may lower lymphedema risk.

## Introduction

Doxorubicin (DOX) is an anthracycline chemotherapeutic agent used as a mainstay treatment for nearly a third of breast cancer patients (Giordano et al., 2012; McGowan et al., 2017). Although its intended mechanism of action is inhibition of the enzyme, topoisomerase 2, to prevent DNA replication and cancer cell division, (Johnson-Arbor and Dubey, 2020), DOX has a number of serious off-target effects including cardiotoxicity and lymphedema that may occur soon after treatment or years later (Norman et al., 2010; Curigliano et al., 2016; Nguyen et al., 2017; Cardinale et al., 2020). For example, DOX increases the risk of arm lymphedema in breast cancer patients subjected to axillary lymph node dissection by nearly 3 –fold (Nguyen et al., 2017), thereby subjecting more breast cancer survivors to a debilitating and irreversible medical condition. In addition to compromised body image, lymphedema predisposes to recurrent infections including severe cellulitis and the lethal cancer, angiosarcoma (Robinson et al., 1991; Cormier et al., 2010; Norman et al., 2010; Ahmed et al., 2011; Bevilacqua et al., 2012; Ridner et al., 2012; Shaitelman et al., 2015; Rivere and Klimberg, 2018).

The treatment of arm lymphedema is limited to compression therapies, manual pumping of the limb, and rarely lymph vessel-to-vein anastomosis (Shaitelman et al., 2015; Rivere and Klimberg, 2018). Accordingly, the search continues for a medication that could protect lymph flow and prevent lymphedema after DOX chemotherapy. In this regard, normal lymph flow relies on the spontaneous and rhythmic contractions of collecting lymph vessels (LVs), which “pump” lymph fluid from the interstitial space to the bloodstream of the systemic circulation to avoid fluid accumulation in peripheral tissues. Disruption of the rhythmic contractions of lymph vessels elevates intraluminal pressure, which can permanently damage the endothelial cells, lymphatic muscle cells (LMCs) and valves that comprise the delicate LVs. The end result is compromised lymph flow (lymphostasis) and development of the clinical manifestation of lymphedema (Zawieja et al., 1991; Ji and Kato, 2001; Nipper and Dixon, 2011; Choi et al., 2015).

In this respect, we recently reported that DOX acutely disrupts the rhythmic contractions of isolated rat mesenteric LVs and attenuates lymph flow *in vivo* at clinically achievable plasma concentrations (Stolarz et al., 2019). DOX appears to impair lymph flow by tonically activating ryanodine receptors (RYRs) to mediate Ca^2+^ release from the sarcoplasmic reticulum of LMCs, thereby causing “calcium leak” and establishing persistently elevated levels of cytosolic free calcium ([Ca^2+^_i_]). Because rhythmic contractions of LMCs rely on the cyclic elevation of [Ca^2+^_i_] to mediate contraction, followed by restoration of resting levels of [Ca^2+^_i_] to permit relaxation, the tonic “calcium leak” induced by DOX disrupts rhythmic contractions and impairs lymph flow (Stolarz et al., 2019). Importantly, we noted that rhythmic contractions in isolated LVs can be restored by blocking RYRs using high concentrations of ryanodine or therapeutic concentrations of dantrolene (DANT). The latter observation may be important, because prophylactic interventions to reduce lymphedema risk associated with DOX chemotherapy are lacking and dantrolene is a clinically available medication that could be repurposed as an anti-lymphedema agent.

Thus, the goal of the present study was to evaluate whether dantrolene (DANT) (Krause et al., 2004; Rosenberg et al., 2015; Ratto and Joyner, 2020), a RYR subtype 1 (RYR1) antagonist approved by the U.S. Food and Drug Administration (FDA) for clinical use, can attenuate DOX-induced “calcium leak” in LMCs and prevent the detrimental effect of DOX on lymph flow *in vivo*. Dantrolene initially was approved by the FDA in 1979 for the prophylactic and acute treatment of malignant hyperthermia, which is caused by excessive Ca^2+^ release mediated by the RYR1 subtype in skeletal muscle cells. It was approved later as a muscle relaxant to alleviate skeletal muscle spasticity. Currently, DANT is available in intravenous and oral formulations (Ratto and Joyner, 2020), with a topical composition recently patented (Henry, 2014). However, DANT has not received attention as an anti-lymphedema therapeutic, largely because the contribution of RYRs to lymph muscle contraction is regarded as minimal and the identity of the RYR subtypes (RYR1, RYR2, and RYR3) expressed by LMCs is unresolved (Atchison and Johnston, 1997; Atchison et al., 1998; Zhao and van Helden, 2003; Jo et al., 2019; Stolarz et al., 2019). Accordingly, the present study also determined whether the RYR1 protein, the therapeutic target for DANT, is expressed by freshly isolated LMCs.

## Methods

### Animals

Rat mesenteric LVs used for *in vitro* studies were isolated from 8 to 12-week-old male and female Sprague-Dawley rats purchased from Envigo RMS (Indianapolis, IN, United States). Animals were deeply anesthetized using 3.5% isoflurane with 1.5 L/min oxygen and euthanized by decapitation. Since no differences in DOX-induced “calcium leak” or abundance of RYR1 protein in LMCs were observed between male and female rats, *in vivo* lymph flow studies were conducted only in female rats. To minimize interference by mesenteric fat during video imaging of LVs *in vivo,* younger (5 to 7-week-old) female Sprague-Dawley rats were purchased from the same vendor (Envigo RMS) as older animals. At the completion of *in vivo* experiments, rats were exsanguinated by cutting the main mesenteric artery under deep anesthesia. All procedures were carried out in accordance with the Guide for the Care and Use of Laboratory Animals as adopted and promulgated by the U.S. National Institutes of Health and approved in animal use protocol #3923 by the Institutional Animal Care and Use Committee at the University of Arkansas for Medical Sciences.

### Diameter Measurement in Cannulated LVs

Second-order collecting LVs (outer diameters, 100–200 μm) were dissected from the rat mesenteric arcade, which was secured in a silicone-lined dish containing physiological salt solution (PSS) composed of (in mmol/L): 119 NaCl, 24 NaHCO_3_, 1.17 NaH_2_PO_4_, 4.7 KCl, 1.17 MgSO_4_, 5.5 glucose, 0.026 EDTA, 1.6 CaCl_2_; bubbled with 7% CO_2_ to maintain pH 7.4. The LVs were cannulated using borosilicate glass micropipettes (outer diameter, 1.2 mm; inner diameter, 0.68 mm) pulled to achieve tip diameters of 75–100 μm (GCP-75–100; Living Systems Instrumentation, Burlington, VT, United States), and then pressurized at 4–5 mm Hg for at least 15 min in a perfusion chamber (Living Systems Instrumentation, Burlington, VT, United States) containing PSS until the appearance of stable patterns of rhythmic contractions. Diameter was monitored through a 10x S Fluor objective with data collected at 15 Hz and analyzed using IonOptix edge-detection software.

### Measurement of Intracellular Free Calcium ([Ca^2+^_i_]) in Cannulated LVs

To measure cytosolic free calcium ([Ca^2+^_i_]) in cannulated LVs, the vessels were prepared as described above, and then incubated in the dark with 2 μmol/L Fura-2 AM (ThermoFisher Scientific Molecular Probes F1221, Waltham, MA, United States) and 0.02% wt/v pluronic acid (Sigma-Aldrich P2443, St. Louis, MO, United States) for 30 min at 37°C, washed with reagent-free PSS and equilibrated for 15 min; then washed again with reagent-free PSS and equilibrated until rhythmic contractions were stable. An inverted Olympus microscope (Olympus Corporation of the Americas, Center Valley, PA, United States) equipped with a 10x S Fluor objective and an IonOptix fluorescence imaging system (IonOptix LLC, Westwood, MA, United States) was used to image LVs loaded with Fura-2 AM. The dye was excited in 50-ms exposures at alternating 340 and 380 nm wavelengths. Fluorescence emission was acquired at 15 Hz and analyzed using IonOptix software.

In order to evaluate the ability of dantrolene (DANT) to prevent RYR-mediated ‘calcium leak’ in LMCs, cytosolic free calcium ([Ca^2+^_i_])-associated fluorescence was recorded in DANT (3 μmol/L) or an equal volume of drug-free dimethyl sulfoxide solvent (1:10,000) for 20 min prior to addition of DOX (10 μmol/L). The [Ca^2+^_i_]-associated fluorescence signal was analyzed after subtraction of background fluorescence at baseline, during the final 5 min of each drug administration, and after drug washout. The [Ca^2+^_i_] value was calculated as the ratio of 340/380 nm wavelengths calibrated to Ca^2+^ standards (ThermoFisher Scientific C3008MP, Waltham, MA, United States).

### High-Speed *In Vivo* Microscopy

Female rats were fasted overnight and anesthetized using 2.5% isoflurane in 1.5 L/min oxygen. A midline abdominal incision was made and a single loop of mesentery was exposed on a customized heated (37°C) chamber filled with HEPES-PSS consisting of (in mmol/L): 119 NaCl, 4.7 KCl, 1.17 MgSO_4_, 1.6 CaCl_2_, 24 NaHCO_3_, 0.026 EDTA, 1.17 NaH_2_PO_4_, 5.5 glucose and 5.8 HEPES); pH was titrated to 7.4 using NaOH. A single LV was visualized and lymph flow was recorded continuously for 10 min to establish a stable baseline. Then dantrolene (10 μmol/L) or an equal volume of dimethyl sulfoxide solvent (1:10,000 dilution) was added to the superfusate bathing the mesentery for 20 min prior to and continuously after tail vein infusion with DOX (10 mg/kg infused over 2 min) or an equivalent volume of saline as control. Lymph flow was recorded continuously prior to and during the 20 min incubation period with DANT and for 60 min after rapid infusion of DOX. Customized software tracked individual lymph cells in flow as described in detail earlier by us (Sarimollaoglu et al., 2018; Stolarz et al., 2019; Garner et al., 2021). Using these data files, flow velocity and positive volumetric flow were calculated to evaluate the effects of DOX and DANT on lymph flow *in vivo*.

### Flow Cytometry

Intact second-order rat mesenteric LVs were dissected free from fat and placed in room-temperature dissociation solution (Solution I) containing (in mmol/L): 145 NaCl, 4 KCl, 1 MgCl_2_, 10 HEPES, 0.05 CaCl_2_, 10 glucose, and 0.5 mg/ml bovine serum albumin; pH was adjusted to 7.4 using NaOH. Subsequently, Solution I was removed carefully with a glass bulb pipette and replaced with 500 µL of new dissociation solution (Solution II) containing Solution I plus papain (1.7 mg/ml, Worthington 3119) and dithioerythritol (1 mg/ml, Sigma D8255) before LVs were incubated for 25–30 min at 37°C. Then, Solution II was removed and replaced with 500 µL Solution III (Solution I plus 1 mg collagenase H (Sigma C8051; 0.5 mg/ml), 1.4 mg collagenase F (Sigma C7926; 0.7 mg/ml), 2 mg trypsin inhibitor (Sigma T9128; 1 mg/ml) and incubated for 5–7 min at 37°C. Immediately following incubation, a glass pipette was used to triturate LVs to release single LMCs from remaining adventitia. Subsequently, 100 µL of 10% FBS was added directly to the suspension containing LMCs and other lymphatic vessel cells, mixed with a pipette, and transferred to a low retention 1.5 ml microcentrifuge tube and spun at 0.9 g for 10 min at 4°C to collect the LMCs. Supernatant was removed and cells were re-suspended in 1 ml of isolation buffer (IB): (phosphate-buffered saline (PBS) containing 0.25% BSA and 2 mM EDTA) and passed through a Falcon® Tube with a 70 μm cell-strainer cap to filter large debris and achieve a single cell suspension. The resuspension step was repeated twice to eliminate residual enzymes from the cell suspension solution. Previous studies by us have shown that this method of LMC isolation produces individual cells for analysis (Garner et al, 2021).

After isolation and washing, the LMCs were fixed using 1% paraformaldehyde dissolved in PBS and rotated on a LabQuake shaker for 20 min at room temperature, protected from light. After 20 min, 900 µL IB was added and tubes were spun at 0.9 g for 25 min. After spinning, the supernatant was removed and pellets were resuspended in blocking buffer (PBS containing 5% BSA, 2 mM EDTA, 0.3% saponin) for 1 h at room temperature. Then cells were divided into groups for incubation with and without primary antibody targeting the RYR1 subtype (Alomone Labs, Jerusalem, Israel; ARR-001) at a dilution of 1:200 and rotated at 4°C overnight. The following morning, the supernatant was removed and cells were resuspended in 1 ml IB with 0.3% saponin and centrifuged at 0.9 g for 25 min at 4°C.

Next, suspensions of LMCs were incubated with Alexa 647 (Abcam, Cambridge, MA, United States; ab150079) at a dilution of 1:100 and smooth muscle specific α-actin-FITC (Abcam, Cambridge, MA, United States; ab8211) at a dilution of 1:100 protected from light for 45 min at room temperature. The cells were rinsed 3 times with IB containing 0.3% saponin before suspension in 300 µL IB for analysis by flow cytometry. Suspensions of LMCs subjected to the same procedure outlined above but without incubation in primary or secondary antibody served as negative controls. Data were collected using the Accuri C6 and LSRFortessa (BD Biosciences, San Jose, CA, United States) in the UAMS Flow Cytometry Core and analyzed with FlowJo-V10 software (FlowJo LLC, Ashland, OR, United States) to measure the protein expression of the RYR1 subtype in isolated LMCs. A separate set of LMCs co-incubated with a competing peptide corresponding to the antigenic sequence for the RYR1 antibody (1:50) was used to evaluate non-specific binding.

### Western Blot

Rat skeletal muscle, heart, and brain were dissected and snap-frozen in liquid nitrogen. Tissues were homogenized using a bullet blender and proteins were extracted with radioimmunoprecipitation assay lysis buffer containing protease inhibitors. Protein concentration was determined with a bicinchoninic acid protein assay (Bio-Rad, Hercules, CA, United States), and 25 µg protein was added to a 2x Laemmli buffer containing β-mercaptoethanol (5%). Gel electrophoresis was performed, and proteins were transferred to a polyvinylidene difluoride membrane (0.22 µm pore size). Protein transfer was confirmed by Ponceau stain of the membrane. The membrane was first incubated in tris-buffered saline (TBS) containing 0.05% Tween-20 and 5% non-fat dry milk to reduce non-specific antibody binding, then incubated overnight at 4°C with primary antibody RYR1 (1:1,000, Alomone Labs, Jerusalem, Israel) prepared in TBS containing 0.05% Tween-20 and 5% non-fat dry milk. After incubation with horseradish peroxidase-conjugated goat anti-rabbit IgG (1:10,000, Cell Signaling Technology, Danvers, MA, United States) at room temperature for 1 h, and washes with TBS-Tween (0.1%), membranes were covered in enhanced chemiluminescence (ECL) Plus Western Blotting Detection Reagent (GE Healthcare Life Sciences, Chicago, IL) and placed on CL-Xposure Film (Thermo Scientific, Waltham, MA, United States). Films were developed and imaged with an AlphaImager® gel documentation system (ProteinSimple, San Jose, CA, United States).

### Chemicals

Doxorubicin HCl injectable USP (Pfizer Inc., obtained from the UAMS Hospital Pharmacy, Little Rock, AR, United States) was stored at 4°C protected from light. Dantrolene (Sigma-Aldrich D9175, St. Louis, MO, United States) was dissolved in DMSO and stored as 10 mmol/L aliquots at -20°C. Fura-2 AM was dissolved in DMSO and stored as 1 mmol/L aliquots at -20°C protected from light. Pluronic F-127 was dissolved in DMSO and stored as 20% (wt/v) aliquots at room temperature.

### Statistics

All data were distributed normally as confirmed using the D’Agostino and Pearson normality test. Data obtained from control and DOX- or DANT-treated preparations at single time points were compared using the unpaired t-test for statistical significance. Comparison of multiple points within a data set was subjected to one-way ANOVA with repeated measures. Comparison between multiple groups with multiple time points was subjected to two-way ANOVA with repeated measures and Tukey multiple comparisons post-test. Data are expressed as Mean ± S.E.M. Differences were judged to be significant at the level of *p* < 0.05.

## Results

### Isolated Lymph Vessels: Intracellular Free Calcium

**FIGURE 1:**
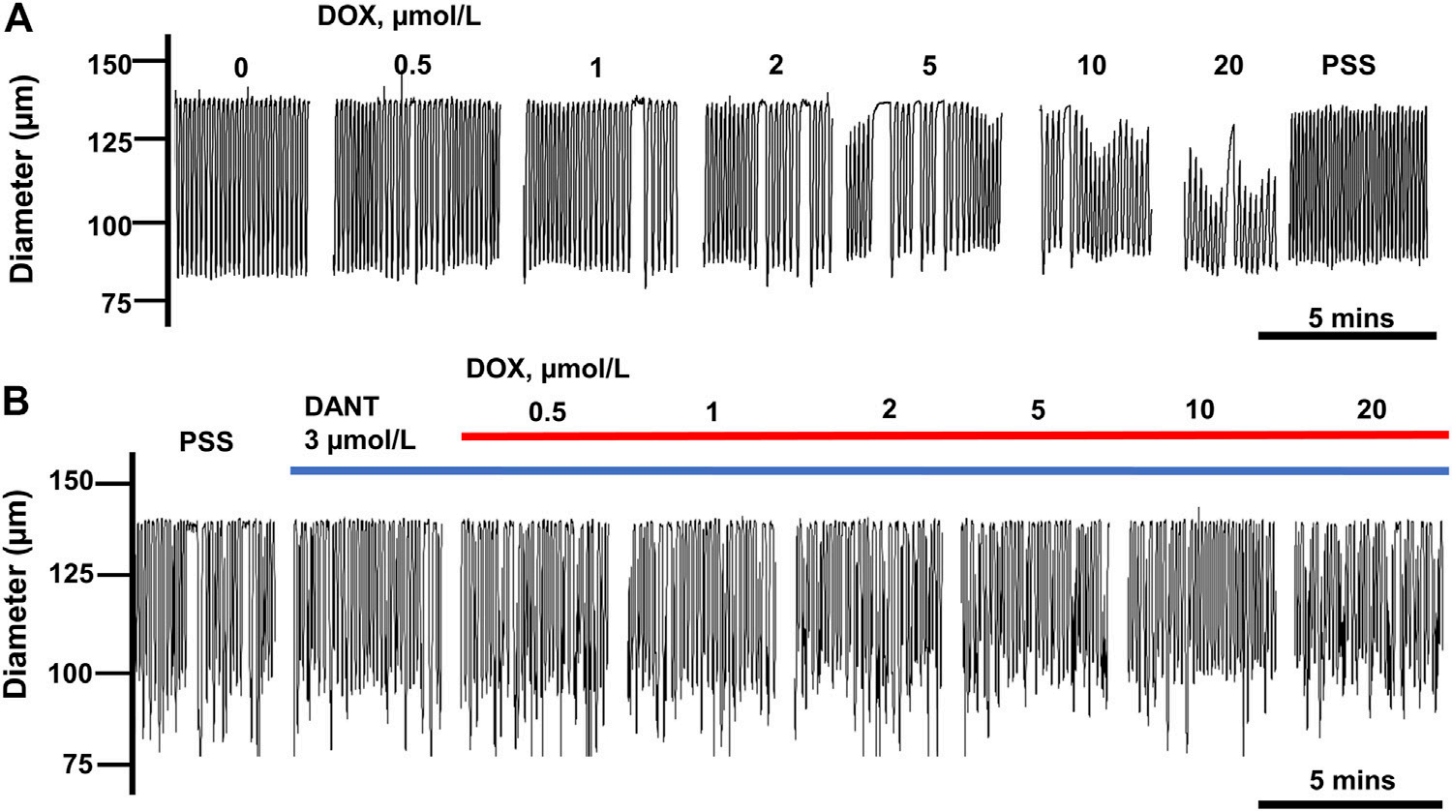
DANT preserves lymphatic contractions in isolated rat mesenteric LVs exposed to DOX. **(A)** Rhythmic contractions of a single LV exposed to increasing concentrations of DOX (0.5–20 μmol/L). DOX progressively inhibited rhythmic contractions and reduced end-diastolic diameter. **(B)** Incubation with DANT (3 μmol/L) prevented DOX-induced inhibition of contractions.

We reported earlier that DOX disrupted rhythmic contractions in cannulated LVs, and this effect was partially prevented by DANT (Stolarz et al., 2019). Because we recently updated our perfusion station, we initially sought to recapitulate these earlier findings here, in order to illustrate the actions of DOX and DANT on lymph vessel contractions as the basis for our current study. Accordingly, rat mesenteric LVs were exposed to increasing bath concentrations of DOX (0.5–20 μmol/L) either in the absence of DANT or 20 min after the addition of DANT (3 μmol/L) to the PSS-filled perfusion chamber. As reported earlier (Stolarz et al., 2019), the cumulative addition of DOX (0.5–20 μmol/L) progressively disrupted the rhythmic contractions of cannulated LVs (Figure 1A). In contrast, the suppression of rhythmic contractions by DOX was effectively blocked by preincubation with DANT (Figure 1B).

**FIGURE 2:**
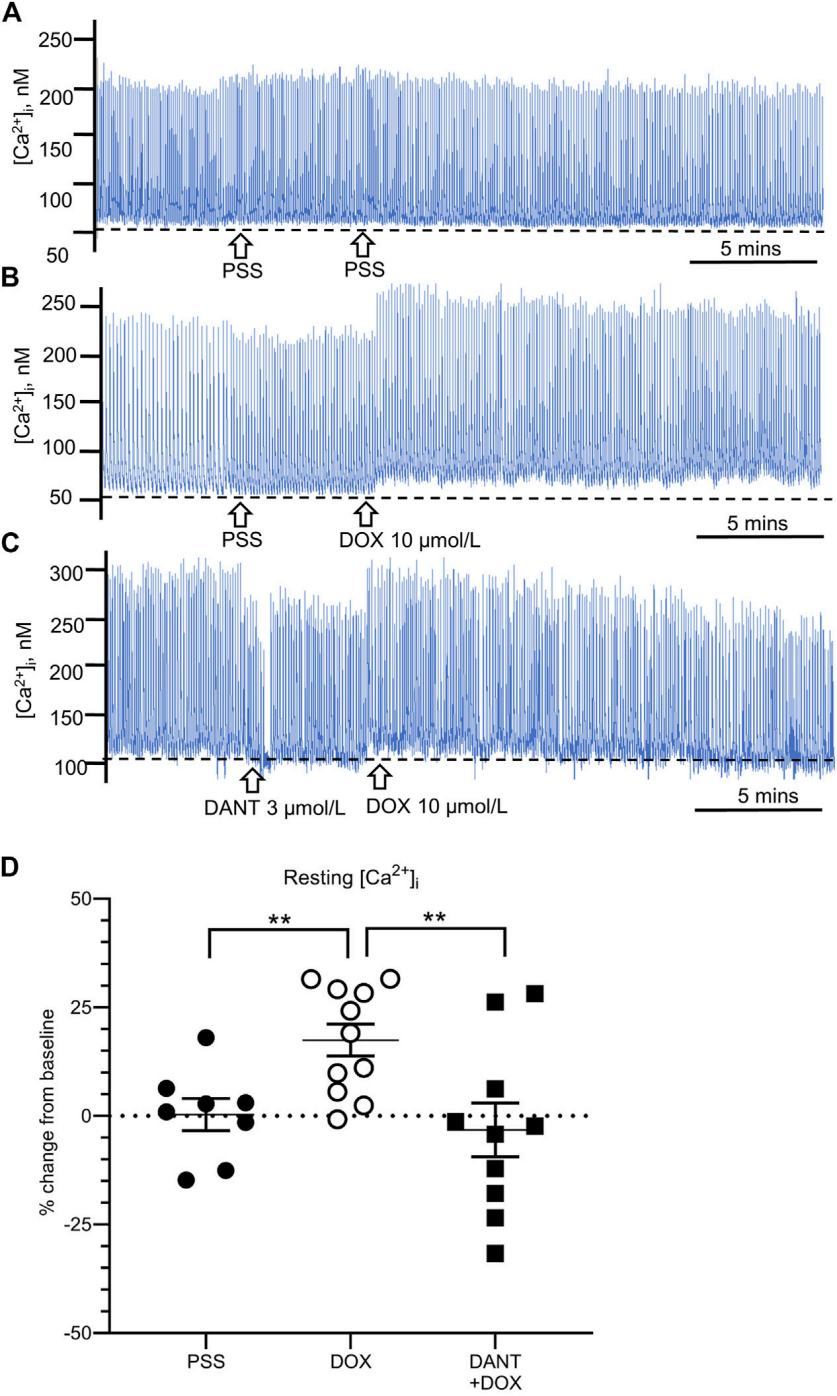
DANT prevents the DOX-induced “calcium leak.” Representative recordings of cytosolic free calcium ([Ca^2+^_i_]) in cannulated LVs loaded with Fura-2. Drug-free solvent, DOX (10 μmol/L), and DANT (3 μmol/L) were added to the bath as indicated by the arrows. The dashed line in each recording represents baseline. **(A)** Drug-free time control. **(B)** Addition of DOX (10 μmol/L) increased resting [Ca^2+^_i_]. **(C)** Incubation with DANT (3 μmol/L) prevented DOX-induced elevation of resting [Ca^2+^_i_] (‘calcium leak’). **(D)** Compared to controls (filled circles), DOX (10 μmol/L; open circles) increased resting [Ca^2+^_i_] at 20 min post-administration, which was prevented by prior incubation with DANT (3 μmol/L; filled squares). Data reported as mean ± S.E.M. and analyzed using unpaired t-test (*n* = 9–11; **p < 0.01).

Next, we used fluorescence capture software to determine whether the mechanism by which DANT prevented the disruption by DOX of rhythmic contractions relied on the ability of DANT to block RYR1 and prevent DOX-induced ‘calcium leak’ from the sarcoplasmic reticulum in LMCs. Cannulated LVs loaded with Fura-2 AM were pre-incubated either in DANT (3 μmol/L) or an equal volume of drug-free solvent for 20 min prior to addition of DOX (10 μmol/L). Resting [Ca^2+^_i_] consistently increased by 17.5 ± 3.7% above baseline in control LVs exposed to DOX (Figures 2B,D–open circles), and this finding was not significantly different between LVs obtained from male rats (*n* = 5) and female rats (*n* = 6) (Supplementary Figure S1A). This finding was not observed in LVs maintained in drug-free solvent as time controls (Figures 2A,D–filled circles). These results corroborated our earlier observation that DOX causes “calcium leak” to tonically elevate [Ca^2+^_i_] in LMCs (Stolarz et al., 2019). However, we show here for the first time that incubation with DANT significantly antagonizes DOX-induced “calcium leak” in cannulated LVs. In these vessels, resting [Ca^2+^_i_] remained stable at 96.7 ± 6.2% of baseline after addition of DOX (Figures 2C,D—filled squares). Interestingly, DANT failed to prevent DOX-induced elevation of [Ca^2+^_i_] in 2 of 11 cannulated LVs, suggesting it failed to antagonize DOX in a minor subset of vessels (Figure 2D—filled squares). The reason for this failure was not readily apparent, and we did not increase DANT concentration progressively because our intention was to use drugs at clinically relevant levels.

### High-Speed *In Vivo* Microscopy

**FIGURE 3:**
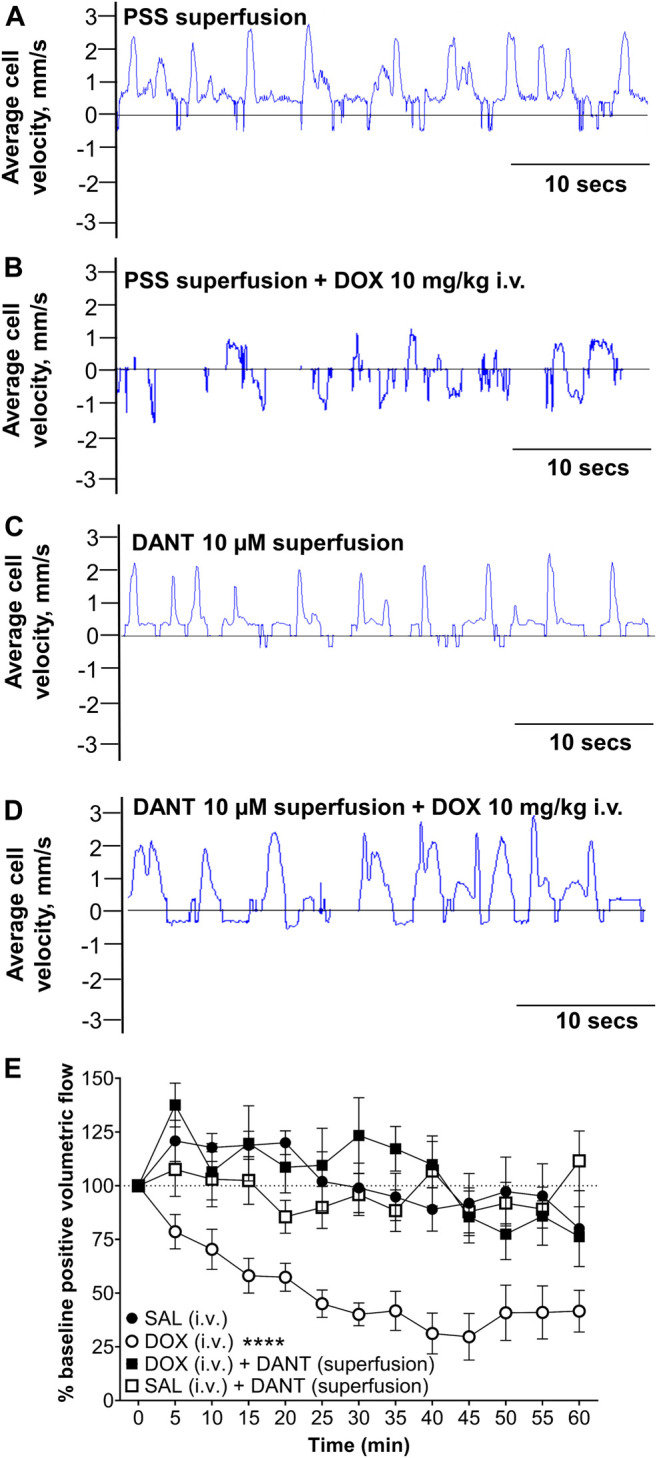
Superfusion of DANT preserves *in vivo* lymph flow in mesenteric LVs of anesthetized rats infused with DOX. Frame-by-frame cell tracking (300 fps video capture) measures the distance travelled by a cell in lymph fluid as a function of time to enable estimation of cell velocity. **(A)** Upward deflections indicate positive (distal-to-proximal) cell velocity in control LVs superfused with PSS. **(B)** Infusion of DOX (10 mg/kg i.v.) reduced average lymph cell velocity to cause “lymphostasis,” which is evident by lower amplitude upward deflections interspersed with downward deflections indicating back flow. **(C)** Superfusion of the mesenteric LVs with DANT (10 μmol/L) had no effect on average lymph cell velocity. **(D)** Additionally, lymph cell velocity was preserved in mesenteric LVs superfused with DANT prior to infusion of DOX (10 mg/kg i.v.). **(E):** Compared with an equi-volume infusion of saline (SAL–filled circles; *n* = 6), systemic administration of DOX (DOX; open circles; *n* = 7) markedly reduced positive volumetric flow in rat mesenteric LVs. Addition of DANT to the superfusate 20 min prior to and continuously after- systemic administration of DOX preserved lymph flow (DOX + DANT; filled squares; *n* = 7). Positive volumetric lymph flow in mesenteric LVs superfused with DANT was stable in animals infused with SAL (SAL + DANT; open squares; *n* = 7). Data reported as mean ± S.E.M. and analyzed using two-way ANOVA (F (3.204, 15.13) = 2.435). **** significant from SAL, DOX + DANT, and SAL + DANT; *p* < 0.0001.

Next, we explored whether the protection offered by DANT against DOX-induced “calcium leak” and disrupted contractions in cannulated LVs would translate to protection against the lymphostasis caused by systemic administration of DOX to anesthetized rats. Accordingly, we measured lymph flow *in vivo* in exposed mesenteric loops of animals subjected to a rapid intravenous infusion of saline (volume control), or subjected to a rapid infusion of DOX at a dose (10 mg/kg i.v.) that establishes steady-state plasma concentrations achieved during clinical chemotherapy protocols (Stolarz et al., 2019). In a subset of the DOX-infused animals, the mesenteric loop containing the LVs was superfused with DANT (10 μmol/L) to provide local block of RYR1 while avoiding potential systemic effects. Using high-speed optical flow cytometry, we calculated volumetric lymph flow in mesenteric LVs by simultaneously tracking vessel diameter and single-cell velocity in lymph fluid (Figures 3A–D; Sarimollaoglu et al., 2018; Stolarz et al., 2019).

Using software developed by us to estimate lymph flow *in vivo* (Sarimollaoglu et al., 2018), the velocity of a single lymph cell is revealed as dynamic and mostly distal-to-proximal in LVs superfused with drug-free PSS (Figure 3A; Supplementary Video S1). The rhythmic upward deflections in a sample recording of cell velocity indicate forward lymph flow and efficient lymphatic pumping enabled by rhythmic contractions (Figure 3A). In contrast, forward cell velocity was markedly reduced at 1 h after rapid infusion of DOX (10 mg/kg i.v.) and occurred bi-directionally (Figure 3B; Supplementary Video S2), indicating impaired lymphatic drainage. To test whether DANT could prevent the impaired lymph flow caused by systemic DOX administration, DANT (10 μmol/L) was maintained in the superfusate bathing the mesenteric loop for 20 min prior to and then after rapid infusion of either DOX (10 mg/kg i.v.) or an equal volume of saline. We chose the 10 μmol/L concentration of DANT, because it corresponds to therapeutic steady-state plasma concentrations (8–12 μmol/L) used in the prophylactic treatment of malignant hyperthermia (Meyler et al., 1979a; Meyler et al., 1979b; Leslie and Part, 1989; Podranski et al., 2005; Hadad et al., 2009). Lymph cell velocity remained dynamic and mostly distal-to-proximal after superfusion of the mesentery with DANT (10 μmol/L), implying that RYR1 do not regulate lymph flow under resting conditions (Figure 3C; Supplementary Video S3). Moreover, when we administered a rapid infusion of DOX (10 mg/kg i.v.) during DANT superfusion of the mesenteric LVs, positive cell velocity in the LVs remained robust and dynamic (Figure 3D; Supplementary Video S4), indicating continued efficiency of lymphatic pumping and preserved distal-to-proximal lymph flow.

Average values for positive volumetric flow under control and experimental conditions are shown in Figure 3E. In control animals infused only with an equi-volume of saline (SAL i.v.) instead of DOX (infusion volume: 1–1.5 ml), positive volumetric lymph flow in mesenteric LVs was not significantly different than baseline during the 60-min study (Figure 3E—filled circles; *n* = 6). In another group of control animals, mesenteric LVs were superfused with DANT for 20 min prior and 1 hour after equi-volume saline infusion. Positive volumetric lymph flow also did not significantly change in response to saline infusion in these LVs when compared to saline infused animals without DANT superfusion, implying that lymph flow was not altered by DANT under basal conditions (Figure 3E—open squares; *n* = 7). In contrast, positive volumetric flow in mesenteric LVs of rats infused with DOX (10 mg/kg i.v.) was markedly reduced to 29.7 ± 10.8% of baseline by 45 min after infusion and flow remained depressed for the remainder of the experiment (Figure 3E—open circles; *n* = 7). Notably, positive volumetric lymph flow was preserved in mesenteric LVs superfused with DANT in DOX-treated animals (Figure 3E—filled squares; *n* = 7). Positive volumetric flow was compared between all groups using two-way ANOVA with repeated measures and Tukey multiple comparisons post-test. DOX treated animals were significantly different from all other groups. No other groups were different from one another. Thus, the local application of DANT effectively prevented DOX-induced reductions in lymph flow.

### Detection of RYR1 in Lymphatic Muscle Cells (LMCs)

**FIGURE 4:**
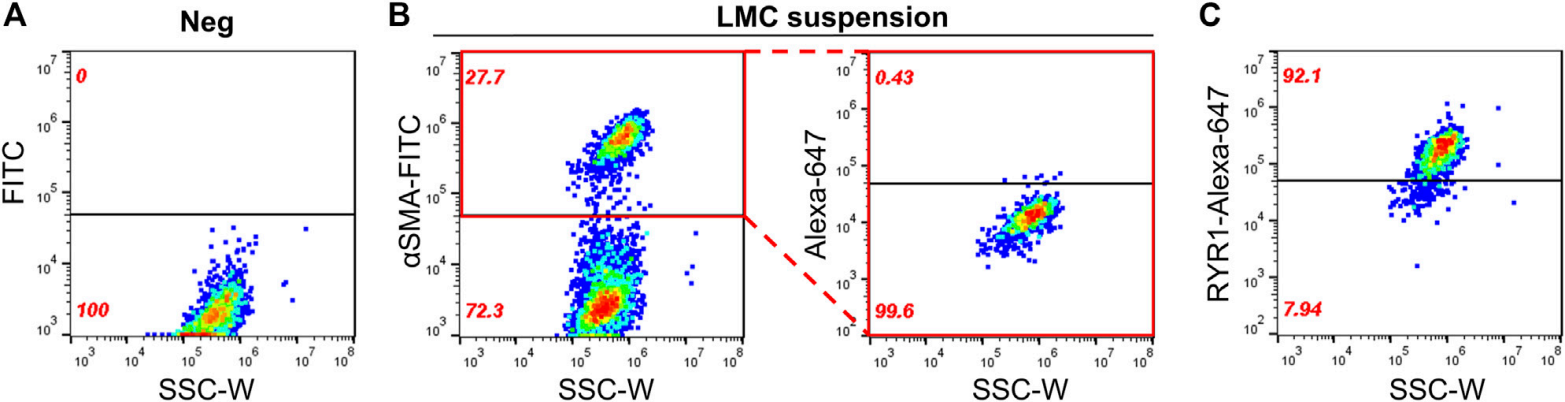
Flow cytometry detects RYR1 in freshly isolated LMCs. Scatter plots of LMC suspensions plotted as side scatter (SSC-W) vs. fluorescent signals. Black horizontal lines denote the gate set. Fluorescent signal appearing above gate indicates positive staining. **(A)** Negative control (Neg) showing background FITC fluorescence of cell population with no antibody staining. Another LMC suspension was used to identify **(B, *left*)** the LMC population stained by α-SMA-FITC (*red box*) and **(B, *right*)** the gate set for non-specific staining of Alexa-Fluor 647 conjugated secondary antibody without primary antibodies within the α-SMA-FITC gated region. **(C)** Positive detection of RYR1 above the Alexa-Fluor 674 gate in LMCs isolated from rat mesenteric LVs. Data representative of five isolations each from male and female rats (*n* = 10).

**FIGURE 5:**
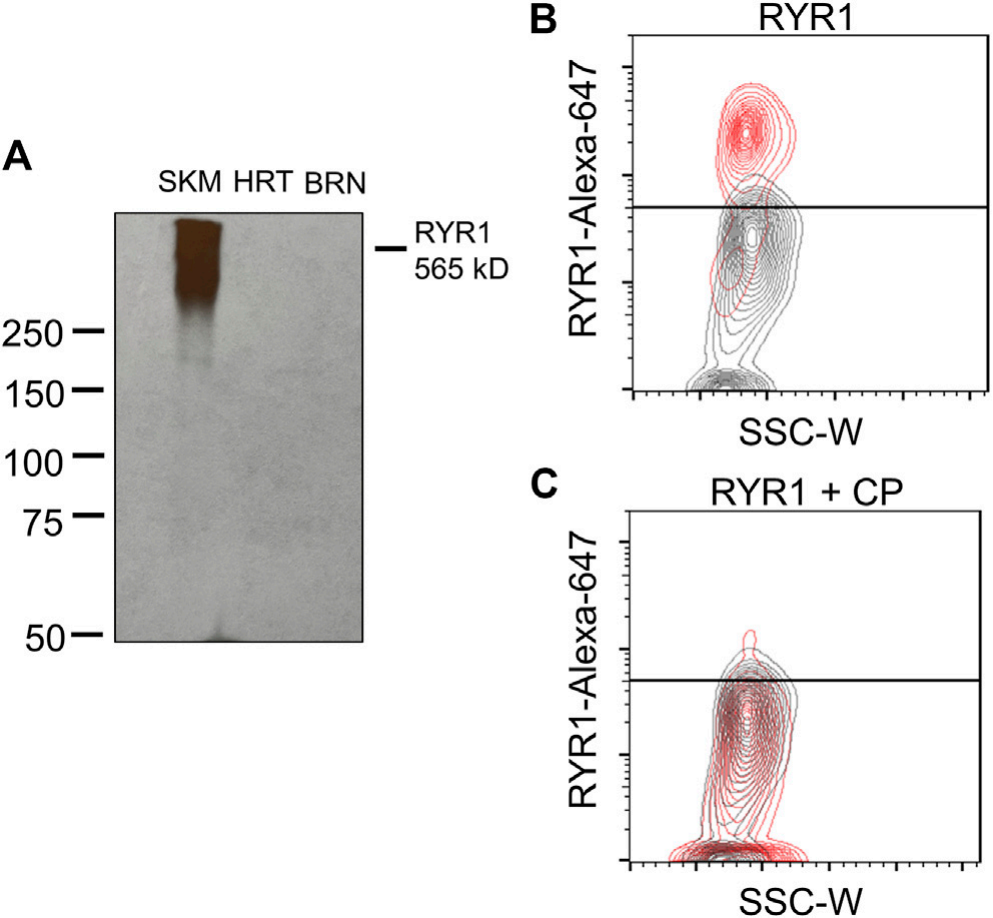
RYR1 antibody selectivity. **(A)** Western blot shows positive detection of RYR1 (∼565 kD) in rat skeletal muscle lysate and no detection in rat heart or brain lysate. **(B,C)** Contour plots of RYR1 antibody testing. **(B)** Red population represents positive detection of RYR1 subtype in male LMCs. Grey population represents the LMC suspension containing no primary antibody. **(C)** No detection in male LMCs after co-incubation with competing peptide (CP; 1:50 dilution) for the RYR1 antibody. Data representative of three isolations for each sex (*n* = 6).

Until now, we have shown that a therapeutic concentration of DANT, a clinical RYR1 antagonist, attenuates DOX-induced “calcium leak” in cannulated LVs and preserves lymph flow in anesthetized rats rapidly infused with DOX to establish a clinically achievable plasma concentration. These findings infer that the RYR1 subtype is functionally expressed in the sarcoplasmic reticulum of rat mesenteric LMCs, a concept not demonstrated definitively to date. Therefore, we sought to detect expression of the RYR1 protein in purified populations of LMCs from male and female rats. Isolated LMCs were co-stained with the smooth muscle marker, α-SMA-FITC, and primary antibody directed against RYR1. Then LMCs were incubated with the secondary antibody conjugated to Alexa-Fluor 647 and subjected to flow cytometry. The FITC signal from LMCs not incubated with antibody (Neg) was used to set the gate to avoid background autofluorescence (Figure 4A; black horizontal line). Another LMC suspension was incubated with α-SMA-FITC and the Alexa-Fluor 647 conjugated secondary antibody in the absence of primary RYR1 antibody. The α-SMA-FITC signal was used to define the gate for the entire LMC population (Figure 4B; left; red box). Within this LMC gated region, the Alexa-647 signal further defined the gate for cells non-specifically stained by the secondary antibody (Figure 4B; right, red box). We used these gates to then measure the Alexa-Fluor 647 signals in the LMC samples additionally incubated with primary antibody for RYR1. Flow cytometry consistently detected immunofluorescence corresponding to the RYR1 protein in single LMCs. Approximately 92.1% of LMCs stained positive for RYR1 expression in populations (*n* = 10 isolations) of α-SMA positive-gated cells isolated from rat mesenteric LVs (Figure 4C). Notably, the α-SMA positive-gated cell populations isolated from mesenteric LVs of female and male rats showed similar proportions of RYR1-expressing cells (Supplementary Figures S1B,C). In further control experiments to confirm antibody specificity, an anti-RYR1 western blot probing rat skeletal muscle (SKM), heart (HRT), and brain (BRN) protein lysates revealed a ∼565 kD immunoreactive band only in skeletal muscle, consistent with the high molecular weight of RYR1 and its known abundance in skeletal myocytes (Figure 5A; Lanner et al., 2010). No signal was observed in lanes loaded with heart and brain lysates, which preferentially express the RYR2 and RYR3 subtypes, respectively (Figure 5A; Lanner et al., 2010). In an additional control study to demonstrate specificity of our RYR antibody for its intended epitope, α-SMA positive LMCs co-incubated with anti-RYR1 and its competing antigenic peptide failed to show RYR1 immunofluorescence (Figures 5B,C).

## Discussion and Conclusion

The present study has three principal new findings. First, the tonic ‘calcium leak’ in LMCs of cannulated rat mesenteric LVs that is caused by DOX-induced opening of RYRs is antagonized by DANT, a clinically available RYR1 antagonist. Thus, we have identified an FDA-approved medication that appears to prevent the DOX-induced cellular event of ‘calcium leak’ that disrupts the cyclic rise and fall of [Ca^2+^_i_] required for lymphatic rhythmic contractions and lymph flow. Second, superfusion of *in-situ* mesenteric LVs with a clinically achievable concentration of DANT prevents the marked lymphostasis caused by a rapid intravenous infusion of DOX, which we have shown establishes therapeutic plasma levels. We interpret this finding to suggest that DANT could be used prophylactically to prevent the disruption of lymph flow caused by DOX, thereby protecting LVs from acute injury and lasting damage. Third, we provide initial evidence that freshly isolated rat mesenteric LMCs express the RYR1 subtype as verification that the recognized target for DANT is a feature of this cell type. Collectively, our findings provide a scientific basis for considering the repurposing of DANT as a therapy that could be given prior to DOX chemotherapy to reduce subsequent lymphedema risk.

For our studies, we chose the rat mesenteric lymphatic bed as our experimental model, since it has been used extensively to characterize lymphatic contractile function and abnormalities of lymph flow. An advantage of this preparation is that the LVs in exposed mesenteric loops are easily imaged for *in-vivo* flow cytometry to measure lymph flow, unlike other more deeply embedded lymphatic beds. Additionally, the extensive networks of LVs in the mesenteric lymphatic circulation permit multiple LVs from a single rat to be removed for contraction and biochemical studies, thereby minimizing animal use and cost (Horstick et al., 2000; Gashev,2002; Galanzha et al., 2005; Galanzha et al., 2007a; Galanzha et al., 2007b; Olszewski and Tárnok, 2008). Our study also addressed sex as a biological variable. Our earlier report described the phenomenon of DOX-induced “calcium leak” in LVs of male rats (Stolarz et al., 2019). Indeed, cancer treatment, including surgery, radiation and chemotherapy, increases the risk of lymphedema for men and women in breast cancer, melanoma, sarcoma, genitourinary, and head and neck tumors (Cormier et al., 2010; Reiner et al., 2011). However, given the health impacts of arm lymphedema with women affected by breast cancer, (Ly et al., 2013), the present study included rats of both sexes for *in-vitro* studies, and then given the finding of no sex differences, the final *in-vivo* studies of lymph flow were conducted only in female animals.

It is important to acknowledge that the precise mechanism by which DOX increases the risk of lymphedema in cancer patients is unclear. The identification of causative factors is complicated by the fact that lymphedema in cancer patients often is delayed and may develop weeks or even years after the final treatment episode (Nguyen et al., 2017; Rivere and Klimberg, 2018). Until recently, it was assumed that the cytotoxic action of DOX damaged the lymphatic circulation, and this effect added to the insults of local surgery and/or radiation, resulted in “multiple hits” and progressive damage to collecting LVs. However, our recent finding that DOX also acts as a reversible pharmacological opener of RYRs in LMCs, implies that DOX can disrupt lymph flow by a mechanism distinct from its cytotoxicity, which also may contribute to the development of lymphedema in already compromised lymphatic circulations. Experimental evidence suggests that elevated intraluminal pressure in LVs caused by disrupted lymph flow can damage the endothelium, muscle cells and delicate valves of collecting LVs, resulting in persistent loss of function (Ji and Kato, 2001). In the present study, we show that a therapeutic concentration of the RYR1 antagonist, DANT, can largely prevent the lymph flow disruption caused by DOX, thereby eliminating a potential contributing factor to lymphedema.

Notably, we designed drug administration protocols for DOX and DANT that were intended to recapitulate the clinical use of these medications. For example, plasma concentrations of DOX attained in patients after rapid infusion in chemotherapy clinics can reach 5 μmol/L (Perez-Blanco et al, 2014), which is within the concentration range that disrupted rhythmic contractions in our rat mesenteric LVs. Additionally, our protocol for rapid intravenous infusion of DOX (10 mg/kg) achieves steady-state plasma concentrations of approximately 0.1 μmol/L similar to clinical practice (de Bruijn et al., 1999; Hempel et al., 2002; Perez-Blanco et al, 2014; Stolarz et al., 2019). Finally, we used concentrations of DANT (3–10 μmol/L) that are similar to the plasma concentrations (∼1–5 μmol/L) that have been observed after prolonged treatment with oral dantrolene for muscle spasticity disorders (Meyler et al., 1981) and plasma concentrations (∼8–12 μmol/L) used in the prophylaxis of malignant hyperthermia (Meyler et al., 1979a; Meyler et al., 1979b; Flewellen et al., 1983; Leslie and Part, 1989; Podranski et al., 2005; Hadad et al., 2009). Thus, at therapeutic concentrations, DANT attenuated DOX -induced “calcium leak” and disruption of rhythmic contractions in the cannulated LVs in our study, and it prevented the detrimental effect of DOX on lymph flow *in vivo*.

In our study, we did not administer DANT systemically, but instead chose to apply it locally to LVs in exposed mesenteric loops. This strategy enabled tight control of the DANT concentration and also avoided the pharmacokinetic and systemic effects that can confound interpretation of drug effects in anesthetized animals. For example, although the biologic half-life of DANT is 4–8 h, oral absorption is variable and slow, and DANT can be converted to a less potent active metabolite in the liver. Additionally, rapid intravenous infusion of DANT has been associated with respiratory distress (Ward et al., 1986; Ratto and Joyner, 2020). Regardless of these complications, it will be important to confirm in future studies that DANT offers protection against DOX-induced lymphostasis when administered systemically. Importantly, the acute prophylactic administration of DANT as a strategy to antagonize the detrimental impact of intravenous DOX on the lymphatic circulation would avoid the risks of hepatitis and pleural effusion associated with chronic treatment. Additionally, it may be possible to administer DANT topically or subcutaneously to at-risk tissues, since in our studies, it appeared to gain access to collecting LVs embedded in the rat mesentery when superfused over the mesenteric loop. Indeed, drug delivery into the subcutaneous space is known to result in rapid lymphatic uptake (Dahlberg et al., 2014; Gao et al., 2020), and a topical formulation of DANT is patented for use in skeletal muscle diseases associated with RYR1 dysfunction (Henry, 2014). This formulation also could be harnessed to protect against localized lymphedema, for example, when applied to the arm to prevent lymphedema after breast cancer surgery.

Studies in skeletal and cardiac myocytes indicate that DOX can bind directly to RYR1 and RYR2 to sensitize Ca^2+^ release (Abramson et al., 1988; Saeki et al., 2002). Of these two RYR subtypes, our data imply that DOX primarily binds to RYR1 in rat mesenteric LMCs since the detrimental effects of DOX on isolated LVs and lymph flow were largely prevented by DANT, an FDA-approved RYR1 antagonist. However, the persisting uncertainty regarding the expression profile and functional role of RYRs in the lymphatic circulation prompted us to directly confirm the expression of the RYR1 protein in LMCs, in order to strengthen our premise that RYR1 is the target for DANT in LMCs that antagonizes the detrimental effects of DOX on the lymphatic circulation. In this regard, transcripts encoding all three RYR subtypes (RYR1, RYR2, and RYR3) have been detected in RNA isolated from human dermal and rat mesenteric LVs. In the latter preparation, the RYR2 and RYR3 proteins were revealed by whole-vessel mount immunostaining, but expression of the RYR1 subtype was not reported (Hasselhof et al, 2016; Jo et al., 2019). In this study, we provide initial direct evidence for the presence of RYR1 in freshly isolated populations of LMCs using a RYR1-specific antibody in flow cytometry.

Several limitations of our study should be acknowledged. First, we did not perform quantitative PCR studies or additional flow cytometry experiments to provide a comprehensive profile of the RYR subtypes expressed by LMCs. Although DANT is a proven RYR1 antagonist, several studies report it also can bind to the RYR3 protein *in vitro* (Zhao et al., 2001; Vaithianathan et al., 2010). Therefore, we cannot definitely state that RYR1 is the sole therapeutic target of DANT in LMCs, although this seems the most likely explanation. Second, lymph flow *in vivo* can be affected by many native variables including endogenous signaling molecules, neurotransmitters, and intravascular volume. It is possible that these and other environmental factors may modulate the efficacy of DANT as an anti-lymphedema medication under conditions less optimal than our controlled *in-vivo* studies (Aukland and Reed, 1993; Zawieja, 2009; Nipper and Dixon, 2011; Choi et al., 2015; Hansen et al., 2015). Third, the short time course of our *in-vivo* studies provided evidence that DANT could protect against the loss of lymph flow caused by rapid DOX infusion. However, in the clinical setting, patients are exposed to multiple doses of DOX over a longer period of time (Brenner et al., 2021). Thus, future studies should measure lymph flow and evaluate lymphatic injury at longer time points after DOX administration and in response to repeated doses of DOX.

In conclusion, our findings imply that DANT, a RYR1 antagonist used to treat malignant hyperthermia and muscle spasticity, is a FDA-approved medication that could be repurposed to prevent the disruption by DOX of the lymphatic rhythmic contractions that propel lymph flow *in vivo*. Additionally, we provide initial and substantial evidence that the RYR1 subtype, which is the recognized therapeutic target of DANT, is expressed in freshly isolated rat mesenteric LMCs. Whereas future studies are required to fully elucidate the long-term benefit of DANT as an anti-lymphedema medication, the findings of the present study provide scientific rationale for further consideration of DANT and other RYR blockers as potential therapeutic agents to protect lymph flow during sessions of DOX chemotherapy.

## Data Availability

The raw data supporting the conclusion of this article will be made available by the authors, without undue reservation.

## References

[B1] Abramson J. J. Buck E. Salama G. Casida J. E. Pessah I. N. (1988). Mechanism of Anthraquinone-Induced Calcium Release from Skeletal Muscle Sarcoplasmic Reticulum. J. Biol. Chem. 263 (35), 18750–18758. 10.1016/s0021-9258(18)37347-2 3198599

[B2] Ahmed R. L. Schmitz K. H. Prizment A. E. Folsom A. R. (2011). Risk Factors for Lymphedema in Breast Cancer Survivors, the Iowa Women's Health Study. Breast Cancer Res. Treat. 130 (3), 981–991. 10.1007/s10549-011-1667-z 21761159 PMC4091732

[B3] Atchison D. J. Johnston M. G. (1997). Role of Extra- and Intracellular Ca^2+^ in the Lymphatic Myogenic Response. Am. J. Physiol. 272 (1), R326–R333. 10.1152/ajpregu.1997.272.1.R326 9039025

[B4] Atchison D. J. Rodela H. Johnston M. G. (1998). Intracellular Calcium Stores Modulation in Lymph Vessels Depends on wall Stretch. Can. J. Physiol. Pharmacol. 76 (4), 367–372. 10.1139/y98-037 9795744

[B5] Aukland K. Reed R. K. (1993). Interstitial-lymphatic Mechanisms in the Control of Extracellular Fluid Volume. Physiol. Rev. 73, 1–78. 10.1152/physrev.1993.73.1.1 8419962

[B6] Bevilacqua J. L. Kattan M. W. Changhong Y. Koifman S. Mattos I. E. Koifman R. J. (2012). Nomograms for Predicting the Risk of Arm Lymphedema after Axillary Dissection in Breast Cancer. Ann. Surg. Oncol. 19 (8), 2580–2589. 10.1245/s10434-012-2290-x 22395997

[B7] Brenner T. Duggal S. Natale J. (2021). Treatment Protocols for Breast Cancer - UpToDate. Availableat: https://www-uptodate-com.libproxy.uams.edu/contents/treatment-protocols-for-breast-cancer?search=treatment protocols for breast cancer&source=search_result&selectedTitle=1∼31&usage_type=default&display_rank=1 (Accessed April 1, 2021).

[B8] Cardinale D. Iacopo F. Cipolla C. M. (2020). Cardiotoxicity of Anthracyclines. Front. Cardiovasc. Med., 7 (7), 26. 10.3389/fcvm.2020.00026 32258060 PMC7093379

[B9] Choi I. Lee S. Hong Y. (2015). The New Era of the Lymphatic System : No Longer Secondary to the Blood Vascular System, Cold Spring Harb Perspect Med. 2,. a006445. 10.1101/cshperspect.a006445 10.1101/cshperspect.a006445 PMC3312397 22474611

[B10] Cormier J. N. Askew R. L. Mungovan K. S. Xing Y. Ross M. I. Armer J. M. (2010). Lymphedema beyond Breast Cancer: a Systematic Review and Meta-Analysis of Cancer-Related Secondary Lymphedema. Cancer 116 (22), 5138–5149. 10.1002/cncr.25458 20665892

[B11] Curigliano G. Cardinale D. Dent S. Criscitiello C. Aseyev O. Lenihan D. (2016). Cardiotoxicity of Anticancer Treatments: Epidemiology, Detection, and Management. CA Cancer J. Clin. 66 (4), 309–325. 10.3322/caac.21341 26919165

[B12] Dahlberg A. M. Kaminskas L. M. Smith A. Nicolazzo J. A. Porter C. J. Bulitta J. B. (2014). The Lymphatic System Plays a Major Role in the Intravenous and Subcutaneous Pharmacokinetics of Trastuzumab in Rats. Mol. Pharm. 11 (2), 496–504. 10.1021/mp400464s 24350780

[B13] de Bruijn P. Verweij J. Loos W. J. Kolker H. J. Planting A. S. Nooter K. (1999). Determination of Doxorubicin and Doxorubicinol in Plasma of Cancer Patients by High-Performance Liquid Chromatography. Anal. Biochem. 266 (2), 216–221. 10.1006/abio.1998.2943 9888978

[B14] Flewellen E. H. Nelson T. E. Jones W. P. Arens J. F. Wagner D. L. (1983). Dantrolene Dose Response in Awake Man: Implications for Management of Malignant Hyperthermia. Anesthesiology 59 (4), 275–280. 10.1097/00000542-198310000-00002 6614536

[B15] Galanzha E. I. Tuchin V. V. Zharov V. P., 2005. *In Vivo* integrated Flow Image Cytometry and Lymph/blood Vessels Dynamic Microscopy. J. Biomed. Opt. 10 (5), 054018. 10.1117/1.2060567 16292978

[B16] Galanzha E. I. Tuchin V. V. Zharov V. P. (2007a). Optical Monitoring of Microlymphatic Disturbances during Experimental Lymphedema. Lymphat Res. Biol. 5 (1), 11–27. 10.1089/lrb.2007.5103 17508899

[B17] Galanzha E. I. Tuchin V. V. Zharov V. P. (2007b). Advances in Small Animal Mesentery Models for *In Vivo* Flow Cytometry, Dynamic Microscopy, and Drug Screening. Wjg 13 (2), 192–218. 10.3748/wjg.v13.i2.192 17226898 PMC4065947

[B18] Gao X. Voronin G. Generaux C. Rose A. Kozhich A. Dalglish G. (2020). Lymphatic Distribution of Etanercept Following Intravenous and Subcutaneous Delivery to Rats. Pharm. Res. 37 (8, 155). 10.1007/s11095-020-02860-6 32720159

[B19] Garner B. R. Stolarz A. J. Stuckey D. Sarimollaoglu M. Liu Y. Palade P. T. (2021). KATP Channel Openers Inhibit Lymphatic Contractions and Lymph Flow as a Possible Mechanism of Peripheral Edema. J. Pharmacol. Exp. Ther. 376 (1), 40–50. 10.1124/jpet.120.000121 33100270 PMC7745085

[B20] Gashev A. A. (2002). Physiologic Aspects of Lymphatic Contractile Function. Ann. N. Y Acad. Sci. 979 (1), 178–187. 10.1111/j.1749-6632.2002.tb04878.x 12543727

[B21] Giordano S. H. Lin Y. L. Kuo Y. F. Hortobagyi G. N. Goodwin J. S. (2012). Decline in the Use of Anthracyclines for Breast Cancer. J. Clin. Oncol. 30 (18), 2232–2239. 10.1200/JCO.2011.40.1273 22614988 PMC3397719

[B22] Hadad G. M. Emara S. Mahmoud W. M. (2009). Development and Validation of a Stability-Indicating RP-HPLC Method for the Determination of Paracetamol with Dantrolene Or/and Cetirizine and Pseudoephedrine in Two Pharmaceutical Dosage Forms. Talanta 79 (5), 1360–1367. 10.1016/j.talanta.2009.06.003 19635371

[B23] Hansen K. C. D'Alessandro A. Clement C. C. Santambrogio L. (2015). Lymph Formation, Composition and Circulation: a Proteomics Perspective. Int. Immunol. 27 (5), 219–227. 10.1093/intimm/dxv012 25788586

[B24] Hasselhof V. Sperling A. Buttler K. Ströbel P. Becker J. Aung T. (2016). “Morphological and Molecular Characterization of Human Dermal Lymphatic Collectors. PLoS One,”. Editor Dettman R W., 11, e0164964. 10.1371/journal.pone.0164964 27764183 PMC5072738

[B25] Hempel G. Flege S. Würthwein G. Boos J. (2002). Peak Plasma Concentrations of Doxorubicin in Children with Acute Lymphoblastic Leukemia or Non-hodgkin Lymphoma. Cancer Chemother. Pharmacol. 49 (2), 133–141. 10.1007/s00280-001-0392-4 11862427

[B26] Henry R. (2014). Topical Composition Comprising Dantrolene And/or Azumolene, WO20141185.

[B27] Horstick G. Kempf T. Lauterbach M. Ossendorf M. Kopacz L. Heimann A. (2000). Plastic Foil Technique Attenuates Inflammation in Mesenteric Intravital Microscopy. J. Surg. Res. 94 (1), 28–34. 10.1006/jsre.2000.5990 11038299

[B28] Ji R. C. Kato S. (2001). Histochemical Analysis of Lymphatic Endothelial Cells in Lymphostasis. Microsc. Res. Tech. 55 (2), 70–80. 10.1002/jemt.1158 11596152

[B29] Jo M. Trujillo A. N. Yang Y. Breslin J. W. (2019). Evidence of Functional Ryanodine Receptors in Rat Mesenteric Collecting Lymphatic Vessels. Am. J. Physiol. Heart Circ. Physiol., 317, H561. 10.1152/ajpheart.00564.2018 31274355 PMC6766729

[B30] Johnson-Arbor K. Dubey R. (2020). Doxorubicin. StatPearls [Internet]. Treasure Island, FL: StatPearls Publishing. Availableat: https://www.ncbi.nlm.nih.gov/books/NBK459232/. 29083582

[B31] Krause T. Gerbershagen M. U. Fiege M. Weisshorn R. Wappler F. (2004). Dantrolene--a Review of its Pharmacology, Therapeutic Use and New Developments. Anaesthesia 59 (4), 364–373. 10.1111/j.1365-2044.2004.03658.x 15023108

[B32] Lanner J. T. Georgiou D. K. Joshi A. D. Hamilton S. L. (2010). Ryanodine Receptors: Structure, Expression, Molecular Details, and Function in Calcium Release. Cold Spring Harb Perspect. Biol. 2 (11, a003996). 10.1101/cshperspect.a003996 20961976 PMC2964179

[B33] Leslie G. C. Part N. J. (1989). The Effect of EU4093 (Azumolene Sodium) on the Contraction of Intrafusal Muscle in the Soleus Muscle of the Anaesthetized Rat. Br. J. Pharmacol. 97 (4), 1151–1156. 10.1111/j.1476-5381.1989.tb12573.x 2790379 PMC1854609

[B34] Ly D. Forman D. Ferlay J. Brinton L. A. Cook M. B. (2013). An International Comparison of Male and Female Breast Cancer Incidence Rates. Int. J. Cancer 132 (8), 1918–1926. 10.1002/ijc.27841 22987302 PMC3553266

[B35] McGowan J. V. Chung R. Maulik A. Piotrowska I. Walker J. M. Yellon D. M. (2017). Anthracycline Chemotherapy and Cardiotoxicity. Cardiovasc. Drugs Ther. 31 (1), 63–75. 10.1007/s10557-016-6711-0 28185035 PMC5346598

[B36] Meyler W. J. Bakker H. Kok J. J. Agoston S. Wesseling H. (1981). The Effect of Dantrolene Sodium in Relation to Blood Levels in Spastic Patients after Prolonged Administration. J. Neurol. Neurosurg. Psychiatry 44 (4), 334–339. 10.1136/jnnp.44.4.334 7241161 PMC490957

[B37] Meyler W. J. Mols-Thürkow H. W. Wesseling H. (1979). Relationship between Plasma Concentration and Effect of Dantrolene Sodium in Man. Eur. J. Clin. Pharmacol. 16 (3), 203–209. 10.1007/BF00562062 499321

[B38] Meyler W. J. Mols-Thurkow I. Scaf A. H. Sargo S. Wesseling H. (1979). The Effect of Dantrolene Sodium on Rat Skeletal Muscle in Relation to the Plasma Concentration. Eur. J. Pharmacol. 53 (4), 335–342. 10.1016/0014-2999(79)90457-6 421731

[B39] Nguyen T. T. Hoskin T. L. Habermann E. B. Cheville A. L. Boughey J. C. (2017). Breast Cancer-Related Lymphedema Risk Is Related to Multidisciplinary Treatment and Not Surgery Alone: Results from a Large Cohort Study. Ann. Surg. Oncol. 24 (10), 2972–2980. 10.1245/s10434-017-5960-x 28766228 PMC5737818

[B40] Nipper M. E. Dixon J. B. (2011). Engineering the Lymphatic System. Cardiovasc. Eng. Technol. 2 (4), 296–308. 10.1007/s13239-011-0054-6 23408477 PMC3568779

[B41] Norman S. A. Localio A. R. Kallan M. J. Weber A. L. Torpey H. A. Potashnik S. L. (2010). Risk Factors for Lymphedema after Breast Cancer Treatment. Cancer Epidemiol. Biomarkers Prev. 19 (11), 2734–2746. 10.1158/1055-9965.EPI-09-1245 20978176 PMC2976830

[B42] Olszewski W. L. Tárnok A. (2008). Photoacoustic Listening of Cells in Lymphatics: Research Art or Novel Clinical Noninvasive Lymph Test. Cytometry A 73A (12), 1111–1113. 10.1002/cyto.a.20654 18985726

[B43] Pérez-Blanco J. S. Fernández de Gatta Mdel. M. Hernández-Rivas J. M. García Sánchez M. J. Sayalero Marinero M. L. González López F. (2014). Validation and Clinical Evaluation of a UHPLC Method with Fluorescence Detector for Plasma Quantification of Doxorubicin and Doxorubicinol in Haematological Patients. J. Chromatogr. B Analyt Technol. Biomed. Life Sci. 955-956 (1), 93–97. 10.1016/j.jchromb.2014.02.034 24631816

[B44] Podranski T. Bouillon T. Schumacher P. M. Taguchi A. Sessler D. I. Kurz A. (2005). Compartmental Pharmacokinetics of Dantrolene in Adults: Do Malignant Hyperthermia Association Dosing Guidelines Work? Anesth. Analg 101 (6), 1695–1699. 10.1213/01.ANE.0000184184.40504.F3 16301243

[B45] Ratto D. Joyner R. W. (2020). Dantrolene. In: StatPearls [Internet]. Treasure Island, FL: StatPearls Publishing. Available at: https://www.ncbi.nlm.nih.gov/books/NBK535398/. 30571019

[B46] Reiner A. S. Jacks L. M. Van Zee K. J. Panageas K. S. (2011). A SEER-Medicare Population-Based Study of Lymphedema-Related Claims Incidence Following Breast Cancer in Men. Breast Cancer Res. Treat. 130 (1), 301–306. 10.1007/s10549-011-1649-1 21735047

[B47] Ridner S. H. Deng J. Fu M. R. Radina E. Thiadens S. R. Weiss J. (2012). Symptom Burden and Infection Occurrence Among Individuals with Extremity Lymphedema. Lymphology 45, 113–123. 23342931

[B48] Rivere A. E. Klimberg V. S. (2018). Lymphedema in the Postmastectomy Patient. Breast January, 514–530. 10.1016/B978-0-323-35955-9.00036-2

[B49] Robinson M. H. Spruce L. Eeles R. Fryatt I. Harmer C. L. Thomas J. M. (1991). Limb Function Following Conservation Treatment of Adult Soft Tissue Sarcoma. Eur. J. Cancer 27 (12), 1567–1574. 10.1016/0277-5379(91)90417-c 1782065

[B50] Rosenberg H. Pollock N. Schiemann A. Bulger T. Stowell K. (2015). Malignant Hyperthermia: a Review. Orphanet J. Rare Dis. 10, 93. 10.1186/s13023-015-0310-1 26238698 PMC4524368

[B51] Saeki K. Obi I. Ogiku N. Shigekawa M. Imagawa T. Matsumoto T. (2002). Doxorubicin Directly Binds to the Cardiac-type Ryanodine Receptor. Life Sci. 70 (20), 2377–2389. 10.1016/S0024-3205(02)01524-2 12150202

[B52] Sarimollaoglu M. Stolarz A. J. Nedosekin D. A. Garner B. R. Fletcher T. W. Galanzha E. I. (2018). High-speed Microscopy for *In Vivo* Monitoring of Lymph Dynamics. J. Biophotonics 11 (8), e201700126. 10.1002/jbio.201700126 29232054 PMC6314807

[B53] Shaitelman S. F. Cromwell K. D. Rasmussen J. C. Stout N. L. Armer J. M. Lasinski B. B. (2015). Recent Progress in the Treatment and Prevention of Cancer-Related Lymphedema. CA Cancer J. Clin. 65 (1), 55–81. 10.3322/caac.21253 25410402 PMC4808814

[B54] Stolarz A. J. Sarimollaoglu M. Marecki J. C. Fletcher T. W. Galanzha E. I. Rhee S. W. (2019). Doxorubicin Activates Ryanodine Receptors in Rat Lymphatic Muscle Cells to Attenuate Rhythmic Contractions and Lymph Flow. J. Pharmacol. Exp. Ther. 371, 278, 289. 10.1124/jpet.119.257592 31439806 PMC6815939

[B55] Vaithianathan T. Narayanan D. Asuncion-Chin M. T. Jeyakumar L. H. Liu J. Fleischer S. (2010). Subtype Identification and Functional Characterization of Ryanodine Receptors in Rat Cerebral Artery Myocytes. Am. J. Physiol. Cel Physiol 299 (2), C264–C278. 10.1152/ajpcell.00318.2009 PMC2928634 20445169

[B56] Ward A. Chaffman M. O. Sorkin E. M. (1986). Dantrolene. A Review of its Pharmacodynamic and Pharmacokinetic Properties and Therapeutic Use in Malignant Hyperthermia, the Neuroleptic Malignant Syndrome and an Update of its Use in Muscle Spasticity. Drugs 32 (2), 130–168. 10.2165/00003495-198632020-00003 3527659

[B57] Zawieja D. C. (2009). Contractile Physiology of Lymphatics. Lymphat Res. Biol. 7 (2), 87–96. 10.1089/lrb.2009.0007 19534632 PMC2925033

[B58] Zawieja D. C. Greiner S. T. Davis K. L. Hinds W. M. Granger H. J. (1991). Reactive Oxygen Metabolites Inhibit Spontaneous Lymphatic Contractions. Am. J. Physiol. 260 (6 Pt 2), H1935–H1943. 10.1152/ajpheart.1991.260.6.H1935 2058726

[B59] Zhao F. Li P. Chen S. R. Louis C. F. Fruen B. R. (2001). Dantrolene Inhibition of Ryanodine Receptor Ca^2+^ Release Channels. Molecular Mechanism and Isoform Selectivity. J. Biol. Chem. 276 (17), 13810–13816. 10.1074/jbc.M006104200 11278295

[B60] Zhao J. van Helden D. F. (2003). ET-1-associated Vasomotion and Vasospasm in Lymphatic Vessels of the guinea-pig Mesentery. Br. J. Pharmacol. 140 (8), 1399–1413. 10.1038/sj.bjp.0705573 14623768 PMC1574159

