# Supplementary material for: Dantrolene Prevents the Lymphostasis Caused by Doxorubicin in the Rat Mesenteric Circulation
Source: Front Pharmacol. 2021 Aug 16;12:727526. doi: 10.3389/fphar.2021.727526 (PMC8415554; doi:10.3389/fphar.2021.727526)
Supplement: Supplementary file 2 [file DataSheet1.pdf]

Dantrolene prevents the lymphostasis caused by doxorubicin in the rat mesenteric circulation

Serena Van[1], Soumiya Pal[1], Brittney R. Garner[2], Kate Steed[1], Vijayalakshmi Sridharan[1], Shengyu Mu[2], Nancy J. Rusch[2], Amanda J. Stolarz[1,2]

Supplemental Figure 1

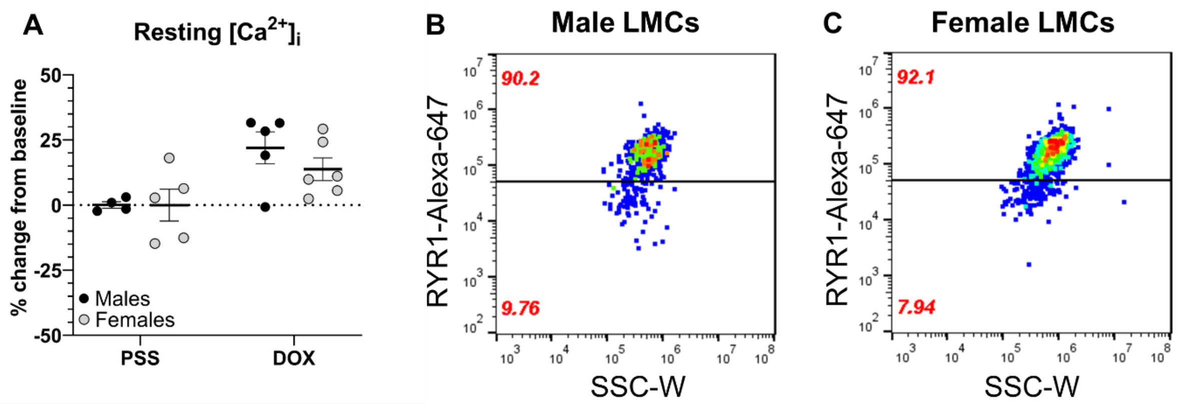

Supplemental Figure 1: Similar response to DOX and RYR1 expression between males and females. (A) DOX (10 µmol/L) induced similar increases in resting $[Ca^{2+}]_i$ at 20 mins post administration in LV isolated from males (black symbols) and female rats (grey symbols). Data presented as mean ± S.E.M (males n=4-5, females n=5-6; p<0.05). **(B-C)** Scatter plots for RYR1 staining in males vs females. Gates (horizontal black line) set based on Figure 4. Similar percent of cells stained positive for RYR1 in LMCs isolated from **(B)** males and **(C)** females. Data representative of 5 isolations for each sex.
